# Policy actors’ perceptions of public participation to tackle health inequalities in Scotland: a paradox?

**DOI:** 10.1186/s12939-023-01869-8

**Published:** 2023-03-30

**Authors:** Neil McHugh, Rachel Baker, Clare Bambra

**Affiliations:** 1 Yunus Centre for Social Business and Health, Glasgow Caledonian University, Glasgow, Scotland UK; 2 Population Health Sciences Institute, Newcastle University, Newcastle, England, UK

**Keywords:** Health inequalities, Health inequities, Public participation, Policy actors, Qualitative, Scotland

## Abstract

**Background:**

Health inequalities are persistent and widening with transformative policy change needed. Radically shifting policy to tackle upstream causes of inequalities is likely to require public participation to provide a mandate, evidence and to address questions of co-design, implementation and acceptability. The aim of this paper is to explore perceptions among policy actors on why and how the public should be involved in policymaking for health inequalities.

**Methods:**

In 2019–2020, we conducted exploratory, in-depth, semi-structured interviews with 21 Scottish policy actors from a range of public sector bodies and agencies and third sector organisations that work in, or across, health and non-health sectors. Data were analysed thematically and used to examine implications for the development of participatory policymaking.

**Results:**

Policy actors viewed public participation in policymaking as intrinsically valuable for democratic reasons, but the main, and more challenging, concern was with how it could affect positive policy change. Participation was seen as instrumental in two overlapping ways: as evidence to improve policies to tackle health inequalities and to achieve public acceptance for implementing more transformative policies. However, our analysis suggests a paradox: whilst policy actors place importance on the instrumental value of public participation, they simultaneously believe the public hold views about health inequalities that would prevent transformative change. Finally, despite broad agreement on the need to improve public participation in policy development, policy actors were uncertain about how to make the necessary changes due to conceptual, methodological and practical challenges.

**Conclusions:**

Policy actors believe in the importance of public participation in policy to address health inequalities for intrinsic and instrumental reasons. Yet, there is an evident tension between seeing public participation as a route to upstream policies and a belief that public views might be misinformed, individualistic, short-term or self-interested and doubts about how to make public participation meaningful. We lack good insight into what the public think about policy solutions to health inequalities. We propose that research needs to shift from describing the problem to focusing more on potential solutions and outline a potential way forward to undertake effective public participation to tackle health inequalities.

## Background

In the UK, the health divide is widening in spite of world-class research on health inequalities and policy recognition [1–3]. A large and widely-accepted evidence base exists on health inequalities and is reported in successive government reports over at least four decades from the Black Report [4] to the Marmot Review [5]. This evidence tells us that those who are worse-off, in terms of socioeconomic position, live shorter lives in worse health than those who are better-off. Importantly, we also know that as health is determined by social, economic and environmental conditions it is possible to effect positive change [6–8]. However, there is incongruity between research and policy and instead of knowledge leading to action that narrows the health divide, it is widening [1]; a trend further exacerbated by the COVID-19 pandemic [9].

### Why has evidence of health inequalities not resulted in effective policy?

Multiple, overlapping theses exist for the disparity between evidence and policy with respect to health inequalities. There is a widely acknowledged ‘lifestyle drift’ where health policy and practice focusses on *downstream* interventions that aim to modify individuals’ health behaviours rather than *upstream* interventions which aim to act on the underlying causes of poor health [10–13]. There are a number of explanations for this drift. Positive health effects are more easily evidenced for short-term downstream interventions than for longer-term structural and redistributive policies that address the upstream causes of health inequalities; the evidence base of the former is thus more developed and easier for policymakers to draw upon than the latter [14–16]. Acting on the underlying causes of poor health is challenging as it requires that non-health sectors, such as environment, social security, education and housing consider health outcomes and necessitates intersectoral policymaking [17, 18]. More insidiously, neoliberal motivations individualise responsibility for poor health and poverty [19] and shift the gaze away from the state as part of the cause and the solution [20].

### A public mandate?

One perceived stumbling block for more transformative policies is the lack of public mandate [21]. Generally, such a mandate relies on public participation through a specific form of representative democracy – voting at the ballot-box. However, depending on electoral systems only to produce long-term policy responses that entail re-thinking and redistribution is unlikely to see health inequalities narrowed. Politicians need to see results quickly due to pressure from short-term electoral cycles, exacerbating the so-called lifestyle drift described above [22–24]. The UK Government’s Levelling-Up white paper [25] is an exemplar of this. Despite an explicit target to reduce health inequalities, as part of tackling regional inequality, the paper is criticised for focusing on an individualistic, medical model of health and health promotion, and diagnostic measures to improve healthy life expectancy [26]. Furthermore, the recent turbulence in UK politics, with three Conservative prime ministers in 2022, the projected reduction in UK public expenditure and the shelving of the planned white paper on health disparities [27] makes the UK Government’s position on tackling health inequalities through Levelling-Up unclear. But perhaps more fundamentally, political manifestos are written to win votes and politicians on the left and right are generally reluctant to take a risk on radical changes (e.g. through taxes or other means to redistribution). At the same time as improving income and health for poorer groups, addressing health inequalities through redistribution is likely to involve some negative impacts (on income and wealth) for richer people and key voter groups. A recent exception is the 2019 Labour Party manifesto that promised radical policy change through increases in public spending and taxation, and nationalisation of key industries, such as energy, water and transport industries [28]. However, the domination of this election by the single issue of Brexit again highlights the limitations of relying solely on electoral systems to reduce health inequalities.

Perhaps recognising these limitations of representative democracy there has been a participatory turn and signs of democratic innovation in some areas of the public sector. Engaging the public is now common within health systems and often recommended to Governments concerned with acting on social determinants of health to reduce health inequalities [29–31]. In the academic literature there is growing recognition that insight into public values for non-health policies and their associated (non-)health outcomes is missing from policy development and has the potential to support upstream policies to tackle health inequalities [32]. Given the long history of academic research into health inequalities, it is perhaps surprising then that we know so little about public perceptions of potential policy solutions. Research evidence and published accounts of consultation and coproduction in practice is limited with the latter also being hard to find. The participatory turn in policymaking means we must consider what is meant by public(s) and how members of the public view and value different policies and solutions to challenge health inequalities.

### Public(s)

*Public(s)* is a protean social construct. There are many different *public(s)* that assemble, or are assembled, for different reasons [33–36]. Within a society, different subsets of people can be identified, sampled, or gather in communities, because of specific characteristics in terms of place, interest, demographics or beliefs [37, 38]. In the construction of *public(s)* for policy or research a key consideration is the type of knowledge or perspective that is deemed relevant for the issue of concern. For example, focusing on lived experience can generate experiential knowledge while acting as citizens can inform broader societal concerns [39, 40]. Thus, different *public(s)* can be drawn upon for different purposes.

### Public views on solutions to health inequalities

From academic literature, only a handful of studies have explored what the public think about potential solutions to health inequalities [41–44]. In Scotland, a Q methodology study explored perspectives on the causes of, and solutions to, health inequalities with a sample of professional stakeholders and community participants and the relationship between perspectives on causes and solutions. Despite community participants recognising structural problems as a cause of health inequalities, structural solutions were not recognised by this same group [42]. Similar findings emerge from a small number of qualitative studies with community groups in Australia [43] and the USA [41]. Putland et al. [43] found that while individuals identified structural and social issues (e.g. unemployment, financial insecurity, level of income, pollution) as the cause of health inequalities, more emphasis was placed on individual behaviours and attitudes than addressing the socio-economic causes. Lundell et al. [41] identified the same, narrow view of individualised, behavioural policy responses in community-based focused groups. In contrast, evidence from a nationally representative survey and two-day citizens’ juries, across three UK cities, found support for proposals aiming to tackle health inequalities through improvement to living and working conditions [44]. However, other macro-economic proposals, such as tax increases, proved controversial with disagreements around the fairness of introducing progressive income taxes and specific details of proposed tax rates and income thresholds influencing discussions.

Insight from public consultation and engagement activities presents a somewhat mixed picture of public views on solutions to health inequalities. In a facilitated discussion as part of Public Health England’s National Conversation on health Inequalities [45] members of the public identified education and early interventions, fair employment and a healthy standard of living as important for tackling health inequalities. The National Institute for Health and Care Excellence (NICE) has undertaken two separate public engagement activities – Citizens Council [46] and Nice Listens [47] – on health inequalities in England. Participants from both activities acknowledged that addressing health inequalities should be a focus of NICE and that where possible the focus should be on prevention rather than treatment. There are contrasting results from two reports of work funded by the Health Foundation. The first, from their COVID-19 impact inquiry [48], found members of the public who participated in workshops, in general, focused on actions that enabled individual choices in three policy areas (education, income and work). However, recent deliberative work prepared for their independent review into health inequalities in Scotland found public panel members initially focused on educational and behavioural solutions but gravitated towards more radical policies, such as higher taxes and greater redistribution, to reduce health inequalities after being presented with expert evidence and other materials [49].

### Why public values and participation?

Generally, rationales for public participation are justified on intrinsic and instrumental grounds [33, 39, 50]. Intrinsic arguments are based around notions of democratic legitimacy: public participation is needed for policy processes to be accountable and transparent and the public should have a say in decisions that affect them, particularly when taxes fund public policies that will affect the public for better or worse. Instrumental arguments centre on making better and more intelligent decisions that result in improved policy outcomes, generating social welfare by allocating resources in a way that increases public value. However, there can exist an “uneasy dance” [51, p. 215] between viewing public participation in policy development as a democratic act or a source of evidence. The goal of public participation has implications for the methods utilised, and a higher-bar, in terms of robustness, is required for public values as evidence (of instrumental worth) vis a vis participation (of intrinsic worth).

Policy actors are those individuals and organisations involved in policy development and debate, they help to construct policy narratives, evidence, and the processes and possibilities for policy implementation [22, 52, 53]. Exploring policy actors’ perceptions on public participation for health inequalities is important because they will influence and inform *how* public participation should be undertaken. Research in the UK suggests a prevailing view among the UK policy community is that the public lack appetite for more egalitarian policies [54]. If this holds then why would policy actors who want to find ways to address health inequalities *seek* public participation, how should participation occur and what tensions exist? The aim of this paper is to explore the perceptions of policy actors in Scotland on why and how the public should participate in policy development to address health inequalities.

## Methods

### Study setting and sampling

The study setting is Scotland. Scotland is an interesting case for two main reasons. First, addressing health inequalities is central to much Scottish Government policy rhetoric (see, for example, the report by the Scottish Government and Local Government on Scotland’s Public Health Priorities [55]). However, despite significant policy attention, Scotland has a lower life expectancy and healthy life expectancy for both males and females when compared with the UK and healthy life expectancy in Scotland is now lower than in 2009–2011 [56, 57]. Second, public participation is a recognised component of the ‘Scottish approach’ to policymaking. The Scottish Parliament is devolved from the UK Parliament, but the division of reserved and devolved powers is complex. Devolved matters include health, economic development, housing, environment and income tax rates. When making and implementing policy in areas of devolved power, the Scottish Government aims for a “consultative and cooperative style” articulated by the Government as the ‘Scottish approach’ to policymaking [58, p. 333, 59]. The approach “aspires to be an exemplar of the New Public Governance, which seeks to be more responsive and creative than classic Public Administration, while being more democratic (participative and collaborative) than the New Public Management paradigm” [60, p. 12]. In Scotland, the 2011 Christie Commission report [61] on the future of public services helped stimulate this shift towards a more participatory approach to policymaking. The Commission recognised the need to move away from top-down public service delivery towards a model “designed with and for people and communities” [61, ix], Examples of participatory policymaking in practice include the Experience Panels set-up to help develop a Scottish social security system [62] and the Community Empowerment (Scotland) Act 2015 that aims to support more collaborative policymaking with communities [63].

Our target sample was policy actors employed within the current policy environment in Scotland, and involved in policy development, decisions and debate affecting health inequalities within the public and third sectors, with a national or local focus. As social, economic and environment factors determine health, policy actors in health and non-health sectors were targeted. Two sampling techniques were used: purposive and snowball sampling. Initially, three keystone participants were identified from within the health sector based on their overarching roles in relation to policy and evidence for health inequalities. These individuals were asked to nominate others who, for example, would offer a different perspective and/or work in a non-health role relevant to the social determinants of health. Participants were recruited via a letter of invitation sent by email alongside an information sheet. Before consenting to be interviewed, participants were given written information about the study and the opportunity to ask questions or seek clarification. Participants were free to withdraw at any time.

### Ethical approval

Ethical approval was obtained from Glasgow School for Business and Society Ethics Committee, Glasgow Caledonian University (reference: GSBS-EC-011).

### Participants

Exploratory, in-depth, semi-structured interviews were conducted with 21 participants across 20 interviews. Eight additional policy actors were approached but either they did not respond, were unavailable or unable to participate due to the civil service code. Participants were from a range of public sector bodies and agencies – Scottish National and Local Government, the NHS (public health) and Health and Social Care Partnerships – and third sector organisations that work in or across health and non-health sectors. Interviewees had a range of roles including developing policy, providing policy advice, undertaking research and advocacy. Given the nature of the sample, only limited descriptive information about participants is provided to preserve anonymity (see Table 1).

**Table 1:** Participant Characteristics

Job Location	Sector			
	Health (H)	Non-Health (NH)	Across health and non-health sectors (HNH)	Total
**Scottish Government (SG)**	4	2	-	6
**Local Government (LG)**	1	3	-	4
**National Health Service (NHS)**	4	-	-	4
**Health and Social Care Partnerships (HSCP)**	-	-	3^*^	3
**Third Sector (TS)**	2	1	-	3
**Total**	11	6	3^*^	**20**

*One interview contained two individuals; this counts as one interview (HSCP-HNH-11).

### Data Collection

Interviews were conducted between October 2019 and December 2020 and lasted approximately one hour. One interview with a Health and Social Care Partnership involved two individuals (see Table 1) with complementing perspectives on the topic. The same questions were posed to both participants who took it in turn to respond and, when relevant, to comment on or add to the other’s response. Interviews were guided by a flexible topic guide based around a number of open-ended questions to guide conversations. The topic guide covered four main areas: priority-setting for health inequalities; across sector policymaking for health inequalities; how health inequalities should be tackled; and, the role of the public in policymaking. The findings reported here draw particularly on the last two of these areas. Interview questions included: regarding the involvement of the public in policy decisions, do you think the public should have a role in policy decisions about tackling health inequalities? Why? What should this role be? If you could introduce one policy to tackle health inequalities what would it be? And why? What do you think needs to happen in order for that policy to be implemented? As public participation to tackle health inequalities will require different *public(s)* for different purposes, we did not prescriptively define *public(s)*; participants were able to refer to different *public(s)* throughout the interviews. Interviews were conducted face-to-face (n = 11), by telephone (n = 8) or by video call (n = 1) and were all audio-recorded. Soundfiles were transcribed verbatim by a transcription company and, following quality checks, were imported into Nvivo 12 [64] for indexing, coding and working towards a thematic structure.

### Analysis

There were different stages of analysis. Initially data driven, descriptive codes were attached to sections of text, and applied systematically through all transcripts, as a means of indexing the data. This approach was similar to open coding [65]. More-focussed coding followed, with superordinate codes introduced to group similar sub-codes together and provide the basis for the development of themes [66]. As data collection and analysis occurred concurrently, we drew on principles of constant comparison [67] to explore and refine themes with participants. We further strengthened themes by considering them in relation to contradictory (or ‘deviant’) cases; this enabled us to refine our interpretation and existing themes, or describe the nature of plurality and introduce new themes [68]. Theoretical coding focussed on concepts from the literature, in particular public participation *as evidence* (of instrumental value) and public participation *as a democratic right* (of intrinsic worth). These themes, alongside those corresponding to how to undertake public participation, structure our findings. This descriptive analysis underpins our discussion of how public participation in policymaking could influence contemporary policymaking practice and what needs to be taken into account when seeking to transform this practice towards more participation in the future. We do so by considering our findings in relation to literature on public understandings of policy solutions for health inequalities, theories of policymaking, methods for eliciting public views and values and policymaking in Scotland.

## Findings

In what follows, we present the findings of our thematic analysis, using the language of our participants where possible to present their perceptions as they articulated them. A notation system is used to indicate who quotes are assigned too. For example, SG-H-2 indicates that the quote comes from participant 2, a Scottish Government participant working in the health sector. We present findings under four main themes: public participation as intrinsically valuable; public participation as instrumentally valuable; the paradox of public participation for policy change; and how to undertake public participation. Each theme begins with a summary of the main findings.

### Public participation as intrinsically valuable for democracy

#### “Yes, of course people should have a voice” (NHS-H-9)

All participants accepted – almost as self-evident – that people should be able to participate in policy development, that good systems and processes include people that are affected by policy, and policy around health inequalities is no different.

Public participation is of intrinsic importance; it is a key part of being in a democracy - *“Democratic accountability … it’s just good per se”* (NHS-H-1). However, the machinery of democratic systems is not sufficient for a well-functioning democracy, and governments and policy actors need to engage with the public outwith election cycles.

*“I think if we don’t involve the public, I mean, how are we policymaking? Who’s making those decisions and who are they for? So, I mean, we’re supposed to live in a democracy, which involves asking people what they think and acting on their views”* (LG-H-17).

Not only is public participation defended on grounds of democratic rights and good procedure, there is a sense that participation is healthy, “*I think the two are really closely linked, having a sort of happy, healthy, inclusive population and a population that feel like they have some sort of say and feel involved in decisions that affect them”* (LG-H-17).

Public participation is generally not done well and there is a “*distance between authority and the public*” (G-H-12). Those in policy are falling short in creating “*an enfranchised and empowered public*” (G-H-12) and “*a massive culture change*” (NHN-H-2), alongside proper investment, is needed so that policy development is more outward looking. But meaningful public participation is expensive.

*“I think we always baulk from investing in democracy in this country so, you know, when we count up how much money we pay councillors, for example, our MSPs kind of… I mean, we can have an argument about how much they’re paid or not but it’s always “Oh it’s a big number, it’s several million pounds, isn’t that a waste of money?” […] democracy is expensive but it’s really important. And if we invested more in it, so if we said, you know, actually part of your democratic duty as a citizen is that one day a year you need to attend… […] you need to … give people a really good opportunity and time-off and whatever to attend or participate in some sort of agenda-setting or prioritisation for some public body or other, that might be a really good investment of the public’s time and involvement”* (NHS-H-2).

### Public participation as instrumentally valuable for policy change

#### “meaningful engagement […] will help the development of better policy that tackles health inequalities (TS-H-10)

All interviewees recognised public participation is needed to effect positive policy change to tackle health inequalities. This manifested in two overlapping sub-themes: public participation as evidence (to design effective policy) and public participation as acceptance (to implement policy).

### Public participation as evidence

#### “it’s the rich source of evidence, rich source of perspective, new source of perspective” (G-H-14)

Participants acknowledge public participation as a legitimate source of evidence that can complement other forms of evidence and, even in its own right, lead to new insights to tackle health inequalities.

Public participation is a way to shift the focus from causes of, towards solutions to, health inequalities. Involving the public could provide insight on how to address health inequalities and on how policies might affect people. In this way the public are a “*rich source of evidence*” and of solutions who could “*throw up a new perspective or a new light on something*” (G-H-14). Their involvement in the policy process could improve policy actors understanding of issues and the potential impact of policy, including unintended consequences, resulting in policy tailored to the needs of ‘the public’.

*“I think, okay, well, we’ve got a lot of qualitative stories and we’ve got a lot of stats describing what the issues are […] but actually no one’s gone and said, okay, what do you want to do about it? So, we know the harrowing stories, we know the statistics, we know how many people are dying, but actually it’s the solutions side of the thing that you’re going to get from the public, so it’s like, okay, what would work for you? So, if we were to put a pot of money in and, say, give you basic income, think about it, what would that do? […] Everything is describing the issues, rather than going, okay, doing X, Y and Z is actually going to help.”* (TS-H-10).

“*I think, well, there’s information about just what people think and their general response so that’s what we generally ask but more useful are what might, the unintended consequences of this might be, so how might you react to this policy, whatever it is, if it was implemented.*” (NHS-H-9).

Those involved in the policy process “*miss things, [and] make mistakes*” (NHS-H-2). Public involvement can challenge assumptions, “*prevent group thinking*” (NHS-H-2) and reduce the risk of introducing policy that is unlikely to help people those most in need. Thus, public participation could lead to better decisions with better outcomes.

*“I think if you really want to understand how to effectively reduce the impact of health inequality or close that gap of health inequality, then we must have lived experience in our policy making processes…it’s almost like understanding the stories behind communities and why they have issues the way they do, you know?”* (G-H-8).

While the public could be an important source of evidence for policy change, public participation does not imply that policy should be guided *only* by what ‘the public wants’, particularly given the diversity of the views likely to emerge. Instead, public participation should be one part of the policy process and “*there will be some areas where the views of the public may not be appropriate*” (G-H-3), such as when technical decisions are required.

*“…you don’t set out, I think, in a conversation to say, “Whatever this group of people tell me, I’m going to take that as read and that’s exactly what I’m going to do.” It’s part of the process and, yeah, it’s a set of opinions, but there’ll be many others to call on too.”* (LG-H-17).

### Public participation as acceptance

#### “So, by that I mean, you know, right from the outset, and thinking about what happens after the policy is written, the democratic process of getting that into action […] often doesn’t happen at the moment” (LG-H-17)

Participants recognise that without having the public on board the chances of making structural changes to introduce more transformative policies to tackle health inequalities are much reduced.

Achieving public acceptance is necessary to implement more preventive and upstream policies to tackle health inequalities. The policies of political parties can shape, and are shaped by, public views. In this way, public acceptance is entwined with political will and acts as a facilitator for policy change: “*to reduce health inequalities requires effort towards common goals”* (LG-H-17) between policymakers and the general public. Developing common goals with the public and “*across services and sectors*” (LG-H-17) can improve the prospects of introducing something that is “*radically different*” as there is “*shared risk*” (HSCP-H-NH-11). The policies needed to tackle health inequalities are centred around the redistribution of income and wealth to target socioeconomic inequalities, such as, the introduction of a Real Living Wage, basic income and changes to taxation and social security policies.

*“…for me it would be about the distribution of income and wealth, so it would need to tap into tax and benefits, it would be income-based…”* (NHS-H-6).

*“…tackling income inequality, ensuring as many people as possible have a properly living wage…”* (NHS-H-2).

Policy actors need to shift policies towards structural approaches with more of a focus on prevention and processes that cut across sectors. These are often outside the reaches of the NHS. Such an approach could prove controversial as it may ultimately entail shifting money from things like hospitals and prisons to investment in early years and economic development or divesting from health services to investing in communities. While public acceptance is not a necessary pre-condition of all policy change, the feasibility of making such (dis)investment decisions can increase if the public are on board: “*you can only govern with the consent of the people you’re governing*” (G-H-12). If the public have bought into why the policy is important and having an increased awareness of the (potential) trade-offs, chances of public protest are reduced and politicians can be less concerned about being viewed as “*that nanny state*” (TS-H-10).

To help achieve these common goals, policy actors and the public need to develop more of a common understanding. Policy actors need to trust the public “*with the uncertainties and the challenges*” (G-H-12) in order to form a more open relationship about how policy is made. Additionally, they should be “*open minded about hearing about other aspects of things that you haven’t considered, and not necessarily writing them off as wrong just because they don’t tie up perfectly with where you’re starting out*” (LG-H-17). Likewise, the public need to recognise that it is not necessarily possible to fund everything and that trade-offs may be required through, for example, increased taxes or shifting resources.

### The paradox of public participation for policy change

#### “another big issue that is about […] public understanding […] there’s a discourse of […] individualism is still very strong and the understanding of health and health inequalities is still very individualistic” (NHS-H-9)

There is an apparent paradox at the heart of public participation. A common belief is the public hold individualistic, behavioural views on health inequalities or are self-interested but that policy development will be improved and the implementation of transformative policies are more likely through public involvement. So before public participation occurs, measures are needed to counteract existing beliefs about health inequalities.

Participants’ recognised the importance of public participation both as a source of new evidence and to help implement, potentially “*radically different*” (HSCP-H-NH-11) policies through their acceptance of them. Simultaneously, there is a commonly held belief that the public, in general, would not suggest radical, upstream, redistributive policies and take a more individualised, behavioural view of health inequalities that does not align with evidence. Together this leads to a kind of paradox, where public participation is required as a mandate for transformative policies, but unlikely to (spontaneously) support those policies.

*“certainly the public understanding of health is, you know, it’s very much about individual behaviour and they don’t see it, they don’t, I don’t think there’s a general understanding that life expectancy, for example, is an end outcome of all of public policy. So maybe that’s the first thing that we need to start, a general understanding that actually life expectancy and inequalities in life expectancy are, they generally are everybody’s business and they’re the outcome of all public policy … probably the NHS plays the smallest part.”* (NHS-H-9).

*“I think that there’s still a community perception that if something’s wrong, I go along to a hospital and get it sorted, as opposed to it being much deeper around how we support our children, how we work as families, how we are more neighbourly, loneliness…”* (HSCP-H-NH-5).

It may be necessary to provide the public with evidence of health inequalities “*so that’s us telling them stuff rather than the other way round*” (NHN-H-2) to change their views. Likewise, if the public do not support a particular policy that should improve health inequalities “*then the question is not to act on it, the question is, how can we communicate with the public in such a way that they appreciate the need for change and come to be supportive of what’s needed for change”* (TS-H-20). For example, to make a wealth tax politically acceptable “*it would need, it would take a sort of shift in what people generally think is how things are, what they generally think is even possible*” (NHN-H-9). This suggests that some members of the public are unaware of the range of possibilities open to tackling health inequalities. This paradox is evident both across participants and also within the same interview (e.g. NHS-H-2 and NHS-H-9) but is not recognised by participants themselves.

The reality of public engagement, as experienced by some participants, informed the belief that the public, particularly those who suffer from health inequalities, hold individualised views of health inequalities: “*a lot of people, especially in lower educational areas, think that fate is probably the most important thing… numbers, aren’t well that’s it… but there’s quite a lot of belief in fate, to move that on, and again that’s quality of education, understanding and… and so on, and that takes a lot to move it from where it is […] I think we’re overwhelmed by people who think fate and fear of foreigners and these are the things outside our control… are driving what’s happening to them and to disabuse that so we’re a long way off that”* (G-H-12). Due to the intersecting vulnerabilities that some people suffer it can be difficult to think beyond the short-term, which might limit attempts at public engagement. Indeed, we might not have a good sense of what they think about certain issues, in particular about shifting resources to prevention, because these types of conversations have not happened.

*“I think a lot of people who bear the brunt of our unequal health inequality spectrum are people who pretty much, by definition whose interests are not being served by the policymaking process and these are people in crappy jobs, in drafty, mouldy houses with no clean, safe local park, crappy transport options, not able to afford food. I mean these are people who the policy system and the economic system beyond that is not serving them […] I think people focus very much on the immediate and [are] not necessarily literate in systems thinking. So, there needs to be conversations around going upstream when they’re just focusing on the manifestation*.” (TS-H-20).

The individualised view of health inequalities that members of the general public are believed to hold is thought to stem from narratives that are perpetuated in society. This is a result of poor communication by organisations that want to tackle health inequalities and from media, politicians and industry who for reasons of self-interest do not want things to change.

*“… a lot of the public narratives and the understandings at the moment aren’t awfully well based on evidence and so that begs questions around what are the power dynamics that make other narratives more powerful. So, kind of individualised narratives around health inequalities or individualised narratives around how people end up in poverty, so in whose interests do those alternative non-evidence-based narratives support? And what media organisations are portraying them in that particular way? And why would they do that? And who are they funded by? And for what interests? And who’s invited to comment on things on news programmes? And who funds them? And to whose interests?”* (NHS-H-2).

*“…that [public understanding of health and health inequalities] will probably take quite a long time to really change […] and of course there’s lots of opposing forces, who don’t want to change that so I think that’s part of it as well, politicians will generally do things that they think will keep them in power or get them in power and will be very reluctant to do anything that’s seen as being too challenging*” (NHS-H-9).

*“I think in consulting people and involving people, yeah, it’s important to recognise people’s different viewpoints and where they’re coming from, but I think some of that’s our responsibility too. So, the way that we talk about inequalities and where they come from and how to perpetuate it, is part of the public narrative about it. And actually, there’s some really good work, I think, some frameworks now that say, you know, “Consider using this kind of language about people being swept away by a tide of poverty and being trapped there.” And trying to reinforce that, you know, it’s not a choice, it’s not an individual choice to be there, that actually it’s the structures that have been responsible that are largely responsible for that.”* (LG-H-17).

Poorer people, facing daily challenges, might not suggest or support upstream structural responses to health inequalities, blaming ‘fate or foreigners’ rather than unjust systems and economic structures. People facing the cognitive distractions and demands of managing to live on a low income, or the uncertainty caused by precarious employment might well not have the headspace to think in the longer-term. However, there are also doubts about what recommendations would emerge from public participation with richer earners, in relation to health inequalities. For redistribution to happen there would be winners and losers.

“*It’s about, you know, so higher earners in Scotland pay more in income tax and for that we get lots more money available for public services. So, I guess that’s… you know, is there an appetite to pay more to get more, better public services, that might be something worth exploring.”* (LG-NH-4).

### How to undertake public participation

#### “I don’t think we’ve been good at getting alongside the public and saying to the public, okay, let’s decide together where it is that we’re going” (HSCP-HNH-7)

For public participation to effect policy change for health inequalities a shift is needed in the policy making process in relation to *when* and *how*.

### Making policy with, rather than for, people

#### “…making policies on behalf of other people I think is pretty misguided sometimes…” (LG-H-17)

Participants view early and longitudinal public participation in policy development as uncontroversial but is something that does not often occur.

Policy decisions are often enacted from the top-down, made on behalf of individuals, or for people, from a “*resource-led*” (TS-H-18) perspective. At best, engagement occurs towards the end of the policy process when most of the work is already undertaken and at worst the public are only informed about decisions after they are made. This approach is “*one of the surest ways of disenfranchising people and antagonising people*” (NHS-H-02).

*“…I think we’re good at saying to the public this is the policy and what do you think?”* (HSCP-HNH-7).

*“Let’s start with how you’re experiencing things and let’s build up from there, instead where we start with is how the system sees it and then we go down to check that against people’s experiences.”* (G-H-3).

Public participation is required at the outset and throughout a policy’s life. While this may not be necessary for all policy decisions, it would be particularly valuable for “*concrete definable decisions where you could bring to bear different sources of evidence for the public to work with”* (NHS-H-1). This would involve providing the public with some sense of the implications of different policies, in terms of impact and cost, and the trade-offs which may exist not only within specific sectors but also in the round. Approaching policy in this way could make it more “*need-led*” (TS-H-18) and “*co-produced*” (HSCP-H-NH-11) with the public helping to “*set the parameters within the debate both in terms of the options and […] the needs and wishes that you want the options to potentially serve*” (NHS-H-01). Policy actors should also inform the public how their evidence, stories and solutions are used, creating a “*feedback loop*” (TS-H-10) that improves their accountability to the public and acknowledges the time and effort the public have put into the process.

*“And so I think a more mature form of public involvement is not about whether or not you shut a particular […] hospital or not, it’s about how do you prioritise across all the different things that you’re doing and balance your budget around that […] people can then make informed decisions about what should the total budget be […] and say, ‘Well, okay, we agree that that should be squeezed’ or ‘Actually I think that demands more money’ but then you have to have the maturity to say, ‘Well where is it coming from, it is tax or is it from some other service?’”* (NHS-H-2).

### Meaningful public participation

#### “it’s an extremely difficult thing to work out how you can actually make it meaningful” (G-NH-15)

Despite broad agreement among participants on the need for a shift in how policy development occurs, this was not accompanied by clear views about how public participation should happen due to conceptual, methodological and practical challenges.

Conceptually, the vastness of health inequalities undermines efforts. The public find it difficult to completely grasp what is being asked of them as health is impacted by multiple policy areas. Methodologically, meaningful public participation should occur where issues can be clearly defined and bodies of evidence presented. For example, the division caused by Brexit is an outcome of poor public participation “*because there’s people casting votes on the basis of not having the full information, of not really understanding the implications, or the implications have not really been spelled out for them.*” (LG-NH-4). A wide array of deliberative and aggregative approaches can be used to elicit the views of the public; but no approach is favoured. Deliberative approaches, such as mini-publics and citizen assemblies and juries are “*effective in getting the public almost to embrace the priority setting decisions*” (NHS-H-1) as participants are given time to consider and discuss evidence, understand trade-offs and change their minds. However, small numbers of individuals involved in these approaches could lead to issues around representativeness and risk biasing decisions towards those with the means and motivation to be involved. How to ensure representation is a potential downside of participatory budgeting and the utilisation of representative groups. While representativeness is less of an issue with public surveys, there is a concern about eliciting meaningful judgements from questions that ask individuals to rank items on a list and that do not get at relative priorities. Finally, on a practical level, there is uncertainty about whether the public are enthusiastic about co-producing policy. Public participation is most likely to occur when a valued community service, such as a local hospital or day centre, is threatened with closure. Public outrage at these decisions often leads to protest from the fear of losing a valued resource. Additionally, undertaking meaningful public participation is a resource intensive exercise, in terms of money, time and effort, which rarely receives the required investment.

*“yes, of course people should have a voice, the question is how do you do that? How do you do that in a way that reaches people with very quiet voices? How do you practically do that when you’ve got so many decisions being made all the time and people with different amounts of knowledge and different interests and different ability to engage? So, I don’t think the question is should you do it, the question is how do you do it and nobody’s got a really good way of doing that I don’t think, you just do your best.”* (NHN-H-9).

*“So, I’d be interested to know what level of involvement the public might want but I think expectations at the moment might be low because people have become used to not being that involved. You only really see a problem where something happens in a community. So classically for the NHS, you know, a local service closes a hospital or whatever or there’s a proposal for that for a ward to close, a children’s ward or something, and suddenly everybody’s up in arms and the public find it really difficult to influence those decisions so that negates that.”* (NHS-H-2).

## Discussion

This paper presents the findings of a qualitative study exploring perceptions on why and how the public should be involved in policy development for health inequalities, among policy actors in Scotland. While public participation is viewed as a necessary part of democracy, the main concern is with public participation as a means to effect positive policy change. This manifests into two overlapping ways: the use of public participation as evidence to improve policies to tackle health inequalities and to achieve public acceptance to implement more transformative policies. To achieve this goal, participants recognise the need for long-term and early engagement with the public and co-production of policy; however, there is uncertainty about how to meaningfully undertake public participation. Our analysis suggests the existence of a paradox. While public participation is recognised as needed to effect positive policy change, simultaneously, a common belief among participants is that the public hold individualised, behavioural (rather than structural) views about health inequalities that mean they may not support upstream solutions. This raises a different kind of tension – telling people what they should think. The emphasis is on convincing the public of the best ways to tackle health inequalities than on eliciting solutions or gaining public acceptance for policy change. In what follows, we discuss our findings in relation to public understandings of policy solutions for health inequalities, theories of policymaking, methods for eliciting public views and values and policymaking in Scotland.

### Why do we need the public for transformative policy change?

The paradox described in our findings raises an important question for public participation: why do we need the public to introduce transformative policies if we need to change their views before they are helpful for policymaking? This view could suggest that policymaking for health inequalities should focus on the intrinsic worth of public participation for democracy rather than its potential instrumental value to improve policy development. However, three issues warrant consideration.

First, failure to embrace other forms of public participation beyond voting at the ballot-box risks leaving it to politicians and elections to determine how to address health inequalities and whether this is a pressing priority. Within Scotland, the limits to devolved powers mean the Scottish Parliament currently lacks the full arsenal of powers to address health inequalities, most notably around employment and social security, and the Scottish Government’s ability to raise money to fund policies is significantly reliant on the complex arrangement of the UK Government block grant and UK spending decisions [69]. However, political inertia from Brexit and the ongoing question of independence are recognised as contributing factors to the lack of progress addressing health inequalities in Scotland [70]. The Scottish Government currently has the means to do more even if it cannot do everything. For example, our findings highlight the importance of progressive taxation policies for reducing health inequalities. While the Scottish Government has made some progress towards a more progressive tax system (compared to the rest of the UK) with changes to income tax, council tax remains a regressive policy despite it being a devolved power [69, 71]. There is a need to move beyond policy rhetoric to policy action. Policy actors recognise that the structures and mechanisms of representative democracy are not sufficient to achieve transformative policy change and are in favour of a participatory approach to policymaking. Despite rhetoric around the participatory nature of policymaking in Scotland, our findings suggest this remains an aspiration rather than an actuality when it comes to complex long-term multi-sectoral issues, like health inequalities, requiring radical change. Given the widening of health inequalities in recent years, improved public participation is one obvious strategy to increase political will to implement more transformative policies.

Second, despite participants’ concerns and hunches that poorer communities and lower educated people might blame ‘fate’ or ‘foreigners’ for poverty and health inequalities (a view that some believe is fuelled by media and concentrations of power and self-interest) and that richer people, if consulted, might not support redistribute policies, we do not know what the public think about key questions around possible solutions to health inequalities. There exists a long-standing body of academic work exploring ‘lay knowledge’ of health; see, for example, reviews by Blaxter [72] and Smith and Anderson [73] and other recent studies [74, 75]. Interestingly, this work shows how conceptions of the causes of poor health are progressing from individual behaviour [72] to more of an understanding of material, environmental and psychosocial pathways [73, 75]. However, as outlined in the introduction, there are only a small number of academic studies exploring public views on potential solutions to health inequalities; only two focus on the UK and have contradictory findings [42, 44]. The findings of McHugh et al. [42], that structural solutions did not resonate with community participants lends support to policy actors’ perceptions that the public hold views about health inequalities that would prevent transformative change. However, the design of this study does not allow for claims about the representativeness of the identified accounts and Smith et al. [44] provide evidence that members of the public would support more upstream policy solutions to address health inequalities. Aspects of study design could help explain these contradictory findings. Q methodology and deliberative approaches both provide stimuli to participants rather than relying on the articulation of responses to open-ended questions that could limit the ideas and insights drawn upon [43]. However, the stimuli are different. Q methodology studies provide participants with a set of statements of opinion, related to the topic in question, to rank order onto a grid (i.e. a card-sort activity), while deliberative approaches provide information and evidence to participants that can cause their views to change. Indeed, most deliberation requires an openness to new information and changing one’s views, with evidence of public views towards health inequalities changing as a result of deliberation [44, 49]. Additionally, the only national survey that exists did not require respondents to make trade-offs between different policies where the (non-)health outcomes and costs are made explicit [44]. Thus, we do not know the relative importance of different policies and/or their outcomes. Taken together this means that we still do not have a good idea about what the public think about policy solutions to health inequalities and policy actors may find it difficult to interpret the limited evidence that exists. We also do not know what, if anything, the general public are willing to sacrifice, in terms of extra taxation, to reduce health inequalities in the UK [32]. In the now well-known words of Rutger Bregman at the World Economic Forum in Davos [76] on a panel about inequality “we’ve got to be talking about taxes. That’s it taxes, taxes, taxes. All the rest is bullshit in my opinion”. This is important as the public may express support for a particular policy but be unwilling to sacrifice anything to see it enacted. Alternatively, the public may not support more transformative policies. But we do not know this and even if such policies are not supported work exploring public views and values would enable us to better understand why.

Third, the evidence base on the specific transformative policies needed to tackle health inequalities is patchy. Umbrella systematic reviews find positive evidence, for example, around regulating the market for tobacco, alcohol and food and for interventions in housing and work environment, but in general, review evidence is low quality, sparse and/or mixed in certain areas, such as social protection and approaches to the economy, which makes it difficult to provide specific recommendations [14, 77, 78]. Such findings may relate to the ‘inverse evidence’ law as described previously. An interesting consequence of this is that when UK health inequality researchers were asked which policies are more likely to reduce health inequalities there were important differences between their personal (expert) opinion and their perception of the strength of available evidence [79]. The former focusing more on socio-economic, redistributive (‘macro’) proposals and the latter more on lifestyle-behavioral interventions. Even though UK health inequality researchers broadly agree that ‘macro’ policies are more likely to reduce health inequalities [79], different policies may impact on (non-)health outcomes in different ways, result in different impacts to different groups of people and substantially differ in cost [80]. This makes it unclear which policy to implement and what trade-offs the public will accept, particularly if there are tax implications.

It is important to note that no single piece of evidence is likely to lead directly to policy change. The wide variety of empirically informed policymaking theories that exist, generally, highlight that a range of factors need to align for change to occur [81]. However, what is clear from the literature, and acknowledged by participants, is that there is missing evidence around what policies to tackle health inequalities the public value. While the policy solutions the public may (not) support remains unclear, public acceptance is viewed by participants, and recognised both within and outwith academia [21, 32, 82] as having the potential to create the political conditions necessary to effect positive policy change to tackle health inequalities.

### How and when to undertake public participation?

The rationale for public participation can impact how it is undertaken. If public participation was solely about democratic reasons the approaches utilised, such as deliberative methods, may become more about “civic education” to reduce the gap between public and expert perspectives (50, p1539) and to understanding how and why public views shift [83]. However, participants think it is more important to have public participation that is instrumentally valuable. This requires longitudinal participation and mixed methods to answer different questions at different levels of abstraction.

Participants’ perceptions on the merits of different approaches to public participation mirrors academic discussions of aggregative, deliberative and participatory methodologies (see, for example, Baker et al. [33], O’Hagan et al. [84] and Parkinson [85]). Aggregative approaches are generally silent when it comes to understanding rationales and reasoning while deliberative and participatory approaches raise questions around representativeness and diversity. No single method provides all the answers and different methodologies are more or less appropriate depending on the question with different questions requiring answers at different points in the policy process.

One way forward for complex issues, such as health inequalities, which are controversial and difficult to resolve, is research combining quantitative data from aggregative approaches with qualitative data from deliberative methods to assess whether potentially transformative policies around income and wealth redistribution, as advocated by participants, are worth their costs to society and are socially legitimate [32, 33]. Aggregative approaches that elicit public values for different transformative policies by presenting the public with trade-offs will provide insight on the direction and intensity of public support. This evidence is currently missing from policy processes. More open discussion of these public values through facilitated public deliberation of informed citizens will provide policy recommendations [33, 86, 87]. This would help counteract participants concerns of the public having individualised, behavioural (rather than structural) views about health inequalities. Instead of telling the public what to think, good deliberative practices entail the reasoned exchange of views among a group of citizens with diverse perspectives, after considering balanced information to arrive at policy recommendations. Such a mixed-method approach would provide policy actors with explicit insight into what the public are willing to trade-off to reduce health inequalities and the reasons behind these values and enable them to take this into account in policy development.

## Conclusion

Policy actors believe in the importance of public participation in policy to address health inequalities for intrinsic and instrumental reasons mirroring discourse in academic literature. Yet, there is a tension evident between seeing public participation as a route to upstream policies and a belief that public views might be misinformed, individualistic, short-term or self-interested and doubts about how to make public participation meaningful. The reality is we do not have a good idea about what the public think about policy solutions to health inequalities. Research needs to shift from describing the problem to focusing more on potential solutions. One way forward, is the combination of aggregative and deliberative approaches to provide insight on public views and values. While public participation is unlikely to be a silver bullet, as a missing source of evidence, it offers the potential to improve current policies and create the political conditions necessary to implement more transformative policies for tackling health inequalities.

## Data Availability

An anonymised dataset is available from the corresponding author on reasonable request.

## References

[1] Marmot M, Allen J, Boyce T, Goldblatt P, Morrison J. Health Equity in England: the Marmot Review 10 years on. The Health Foundation; 2020. 10.1136/bmj.m693 32094110

[2] Scottish Government. Long-term Monitoring of Health Inequalities: January 2020 report.Health and Social Care; 2020. (An Official Statistics publication for Scotland).

[3] Smith KE, Bambra C, Hill SE. Health Inequalities: critical perspectives. Health Inequalities. Oxford University Press; 2015. 27748106

[4] Department of Health and Social Security (1980). Inequalities in Health.

[5] Marmot M. Fair Society: Healthy Lives. Strategic Review of Health Inequalities in England Post-2010. 2010. (The Marmot Review).

[6] Dahlgren G, Whitehead M. The Dahlgren-Whitehead model of health determinants: 30 years on and still chasing rainbows. Public Health. 2021 Oct;199:20–4. 10.1016/j.puhe.2021.08.009 34534885

[7] Dahlgren G, Whitehead M (1991). Policies and strategies to promote social equity in health.

[8] Kelly-Irving M, Ball WP, Bambra C, Delpierre C, Dundas R, Lynch J et al. Falling down the rabbit hole? Methodological, conceptual and policy issues in current health inequalities research.Critical Public Health. 2022 Feb7;1–11.

[9] Bambra C, Lynch J, Smith KE. The unequal pandemic. COVID-19 and Health Inequalities. Policy Press Shorts Insights; 2021.

[10] Graham H. Health inequalities, social determinants and public health policy. policy polit. 2009 Oct 1;37(4):463–79.

[11] Hunter DJ, Popay J, Tannahill C, Whitehead M, Elson T. Learning lessons from the past: shaping a different future. Marmot Review Working Committee 3 Cross-cutting sub-group report. 2009.

[12] Katikireddi SV, Higgins M, Smith KE, Williams G. Health inequalities: the need to move beyond bad behaviours: Table 1. J Epidemiol Community Health. 2013 Sep;67(9):715–6. 10.1136/jech-2012-202064 23486925

[13] Phillips C, Fisher M, Baum F, MacDougall C, Newman L, McDermott D. To what extent do australian child and youth health policies address the social determinants of health and health equity?: a document analysis study. BMC Public Health. 2016 Dec;16(1):512. 10.1186/s12889-016-3187-6 PMC4908806 27301393

[14] Bambra C, Gibson M, Sowden A, Wright K, Whitehead M, Petticrew M. Tackling the wider social determinants of health and health inequalities: evidence from systematic reviews.Journal of Epidemiology & Community Health. 2010 Apr1;64(4):284–91. 10.1136/jech.2008.082743 PMC2921286 19692738

[15] Ogilvie D. Systematic reviews of health effects of social interventions: 2. Best available evidence: how low should you go? J Epidemiol Community Health. 2005 Oct;59(1):886–92. 10.1136/jech.2005.034199 PMC1732915 16166365

[16] Petticrew M. Evidence for public health policy on inequalities: 1: The reality according to policymakers. Journal of Epidemiology & Community Health. 2004 Oct 1;58(10):811–6. 10.1136/jech.2003.015289 PMC1763325 15365104

[17] Solar O, Valentine N, Rice M, Albrecht. Moving Forward to Equity In Health: What kind of intersectoral action is needed? An approach to an intersectoral typology. In Nairobi, Kenya; 2009.

[18] WHO. Intersectoral governance for health in all policies - structures, actions and experiences. World Health Organization on behalf of the European Observatory on Health Systems and Policies; 2012.

[19] Collins C, McCartney G, Garnham L. Neoliberalism and health inequalities. Health Inequalities: Critical Perspectives.Oxford University Press; 2016.

[20] Robinson T, Brown H, Norman PD, Fraser LK, Barr B, Bambra C. The impact of New Labour’s English health inequalities strategy on geographical inequalities in infant mortality: a time-trend analysis. J Epidemiol Community Health. 2019 Jun;73(6):564–8. 10.1136/jech-2018-211679 30890592

[21] Mackenbach JP (2011). Can we reduce health inequalities? An analysis of the English strategy (1997–2010). J Epidemiol Community Health (1979-).

[22] Carey G, Crammond B. Action on the social determinants of health: views from inside the policy process. Soc Sci Med. 2015 Mar;128:134–41. 10.1016/j.socscimed.2015.01.024 25616195

[23] Douglas M, Smith KE, Bambra C (2016). Beyond ‘health’: why don’t we tackle the cause of health inequalities?. Health Inequalities: critical perspectives.

[24] Exworthy M. Policy to tackle the social determinants of health: using conceptual models to understand the policy process.Health Policy and Planning. 2008 Jul22;23(5):318–27. 10.1093/heapol/czn022 18701553

[25] HM Government. Levelling up the United Kingdom. Secretary of State for levelling up, Housing and Communities. HM Government; 2022.

[26] Ralston R, Smith K, O’Connor CH, Brown A. Levelling up the UK: is the government serious about reducing regional inequalities in health? BMJ. 2022 Jun 1;e070589. 10.1136/bmj-2022-070589 35649577

[27] Iacobucci G. Health inequalities: Government must not abandon white paper, health leaders urge.BMJ. 2022;378. 10.1136/bmj.o2366 36180076

[28] The Labour Party. It’s time for real change. The Labour Party Manifesto 2019. Labour; 2019.

[29] Healthcare Improvement Scotland. Healthcare Improvement Scotland - Community Engagement. Community Engagement. 2022.

[30] NHS England. Patient and Public Participation in Commissioning Health and Care: Statutory Guidance for Clinical Commissioning Groups and NHS England. 2019.

[31] WHO. Closing the gap in a generation: health equity through action on the social determinants of health. Final report of the commission on Social Determinants of Health. World Health Organisation. Commission on Social Determinants of Health; 2008.

[32] McHugh N (2022). Eliciting public values on health inequalities: missing evidence for *policy windows* ?. Evid Policy.

[33] Baker R, Mason H, McHugh N, Donaldson C. Public values and plurality in health priority setting: what to do when people disagree and why we should care about reasons as well as choices. Volume 113892. Social Science & Medicine; 2021 Apr. 10.1016/j.socscimed.2021.113892 PMC8135121 33882440

[34] Barnett C. Convening Publics: The Parasitical Spaces of Public Action. The SAGE Handbook of Political Geography. United Kingdom:SAGE Publications Ltd; 2008.pp. 403–18.

[35] Escobar O. Pluralism and Democratic Participation: What Kind of Citizen are Citizens Invited to be? Contemporary Pragmatism.2017 Nov17;14(4):416–38.

[36] Stewart E (2016). Publics and their Health Systems.

[37] Brunton G. Narratives of community engagement: a systematic review-derived conceptual framework for public health interventions. 2017;15. 10.1186/s12889-017-4958-4 PMC5725895 29228932

[38] Popay J (2006). Community engagement for health improvement: questions of definition, outcomes and evaluation. A background paper prepared for NICE.

[39] Fredriksson M, Tritter JQ (2017). Disentangling patient and public involvement in healthcare decisions: why the difference matters. Sociol Health Illn.

[40] Stewart E, Smith-Merry J, Geddes M, Bandola-Gill J. Opening up evidence-based policy: exploring citizen and service user expertise. Evid Policy. 2020 May;16(1):199–208.

[41] Lundell H, Niederdeppe J, Clarke C. Public views about Health Causation, Attributions of responsibility, and Inequality. J Health Communication. 2013 Sep;18(9):1116–30. 10.1080/10810730.2013.768724 23679219

[42] McHugh N, Baker R, Biosca O, Ibrahim F, Donaldson C. Who knows best? A Q methodology study to explore perspectives of professional stakeholders and community participants on health in low-income communities. BMC Health Serv Res. 2019 Dec;19(1):35. 10.1186/s12913-019-3884-9 PMC6332861 30642316

[43] Putland C, Baum FE, Ziersch AM. From causes to solutions - insights from lay knowledge about health inequalities. BMC Public Health. 2011 Dec;11(1):67. 10.1186/1471-2458-11-67 PMC3038904 21281478

[44] Smith KE, Macintyre A, Weakley S, Hill SE, Escobar O, Fergie G. Public understandings of potential policy responses to health inequalities: evidence from a UK national survey and citizens’ juries in three UK cities. Social Science & Medicine; 2021. Oct;114458. 10.1016/j.socscimed.2021.114458 PMC8711040 34655938

[45] Public Health England. National Conversation on Health Inequalities. Report of event held on 25 June 2014. Public Health England; 2014.

[46] Nice Citizens Council. Inequalities in Health. National Institute for Health and Clinical Excellence; 2006.

[47] NICE. NICE listens: public dialogue on health inequalities. National Institute for Health and Care Excellence; 2022. (NICE Listens). 39190727

[48] Suleman M, Sonthalia S, Webb C, Tinson A, Kane M, Bunbury S et al. Unequal pandemic, fairer recovery. The COVID-19 impact inquiry report.The Health Foundation; 2021.

[49] Diffley Partnership. Health Inequalities in Scotland: Public Engagement Research. 2022. (Report by The Diffley Partnership, prepared for The Health Foundation).

[50] Tenbensel T. Virtual special issue introduction: public participation in health policy in high income countries – A review of why, who, what, which, and where? Soc Sci Med. 2010 Nov;71(9):1537–40. 10.1016/j.socscimed.2010.08.005 20869799

[51] Pallett H. The new evidence-based policy: public participation between ‘hard evidence’ and democracy in practice. evid policy. 2020 May 1;16(2):209–27.

[52] Shiffman J, Smith S. Generation of political priority for global health initiatives: a framework and case study of maternal mortality.Health Policy. 2007;370. 10.1016/S0140-6736(07)61579-7 17933652

[53] Littleton C, Star C, Fisher M, Ward PR (2022). Policy actors’ perceptions on applying a SDH approach in child health policy in Australia: a cross-disciplinary approach (public health and political science). Australian J Public Adm.

[54] Smith KE. Beyond evidence-based policy in Public Health: the interplay of ideas. Palgrave Macmillan UK; 2013.

[55] Scottish Government. Public Health Priorities for Scotland. Population Health Directorate: scottish government. COSLA.; 2018. p. 52.

[56] NRS. Healthy life expectancy 2019–2021. National Records of Scotland; 2022.

[57] ONS. Health state life expectancies, UK: 2017 to 2019. Office for National Statistics; 2021.

[58] Cairney P, Russell S, St Denny E. The ‘Scottish approach’ to policy and policymaking: what issues are territorial and what are universal? Policy & Politics. 2016 Jul;44(3):333–50.

[59] Housden P. This is us: a perspective on public services in Scotland. Public Policy and Administration. 2014 Jan;29(1):64–74.

[60] What Works Scotland. Key Messages About Public Service Reform in Scotland. 2019.

[61] Christie Commission (2011). Commission on the future delivery of Public Services.

[62] Scottish Government. Social Security Experience Panels: how to get involved. 2019.

[63] Steiner A, McMillan C, Hill O’Connor C. Investigating the contribution of community empowerment policies to successful co-production- evidence from Scotland. Public Manage Rev. 2022 Feb;3:1–23.

[64] QSR International. Nvivo 12. 2018.

[65] Strauss A (1987). Qualitative analysis for social scientists.

[66] Braun V, Clarke V. Using thematic analysis in psychology. Qualitative Res Psychol. 2006 Jan;3(2):77–101.

[67] Glaser B, Strauss A. The Discovery of grounded theory: strategies for qualitative research. Transaction Publishers; 1967.

[68] Seale C. The quality of qualitative research. SAGE Publications; 1999.

[69] Hawkey D, Smith C, Whyte P. Tackling the social determinants of health inequalities: a review of scottish powers. IPPR Scotland; 2022. (The Health Foundation. Independent Review into Health Inequalities in Scotland).

[70] Finch D, Wilson H, Bibby J. Leave no one behind: the state of health and health inequalities in Scotland. The Health Foundation; 2023.

[71] IFS. Scottish Budget changes to tax and benefit system widen gap with rest of the UK, with higher taxes and more redistribution to poorer families. Institute for Fiscal Studies; 2023.

[72] Blaxter M. Whose fault is it? People’s own conceptions of the reasons for health inequalities. Soc Sci Med. 1997 Mar;44(6):747–56. 10.1016/s0277-9536(96)00192-x 9080559

[73] Smith KE, Anderson R (2018). Understanding lay perspectives on socioeconomic health inequalities in Britain: a meta-ethnography. Sociol Health Illn.

[74] Garthwaite K, Bambra C. ‘It’s like being in Tattooville’: An ethnographic study of territorial stigma and health in a post-industrial town in the North East of England. Health & Place. 2018 Nov;54:229–35. 10.1016/j.healthplace.2018.10.005 30388457

[75] Garthwaite K, Bambra C. How the other half live”: Lay perspectives on health inequalities in an age of austerity. Soc Sci Med. 2017 Aug;187:268–75. 10.1016/j.socscimed.2017.05.021 PMC5529211 28511818

[76] The Guardian. Rutger Bregman tells Davos to talk about tax: “This is not rocket science”. 2019.

[77] Hillier-Brown F, Thomson K, Mcgowan V, Cairns J, Eikemo TA, Gil-Gonzále D et al. The effects of social protection policies on health inequalities: Evidence from systematic reviews. Scand J Public Health. 2019 Aug 1;47(6):655–65. 10.1177/1403494819848276 31068103

[78] Naik Y, Baker P, Ismail SA, Tillmann T, Bash K, Quantz D, et al. Going upstream – an umbrella review of the macroeconomic determinants of health and health inequalities. BMC Public Health. 2019 Dec;19(1):1678. 10.1186/s12889-019-7895-6 PMC6915896 31842835

[79] Smith KE, Kandlik Eltanani M. What kinds of policies to reduce health inequalities in the UK do researchers support? J Public Health. 2015 Mar;37(1):6–17. 10.1093/pubmed/fdu057 PMC4340326 25174045

[80] Richardson E, Fenton L, Parkinson J, Pulford A, Taulbut M, McCartney G et al. The effect of income-based policies on mortality inequalities in Scotland: a modelling study.The Lancet Public Health. 2020Mar;5(3):e150–6. 10.1016/S2468-2667(20)30011-6 32113518

[81] Smith KE, Katikireddi SV. A glossary of theories for understanding policymaking. J Epidemiol Community Health. 2013 Feb;67(2):198–202. 10.1136/jech-2012-200990 23100379

[82] Health Foundation. Building public understanding of health and health inequalities - The Health Foundation. 2022.

[83] Reckers-Droog V, Jansen M, Bijlmakers L, Baltussen R, Brouwer W, van Exel J. How does participating in a deliberative citizens panel on healthcare priority setting influence the views of participants? Health Policy. 2020 Feb 1;124(2):143–51. 10.1016/j.healthpol.2019.11.011 31839335

[84] O’Hagan A, MacRae C, O’Connor CH, Teedon P. Participatory budgeting, community engagement and impact on public services in Scotland. Public Money & Management. 2020 Aug;17(6):446–56.

[85] Parkinson J. Legitimacy problems in deliberative democracy. Polit Stud. 2003 Mar;51(1):180–96.

[86] Abelson J, Blacksher EA, Li KK, Boesveld SE, Goold SD. Public Deliberation in Health Policy and Bioethics: Mapping an emerging, interdisciplinary field.Journal of Public Deliberation. 2013;9(1).

[87] Blacksher E, Diebel A, Forest PG, Goold SD, Abelson J. What Is Public Deliberation? Hastings Center Report. 2012 Mar;42(2):14–6. 10.1002/hast.26 22733324

